# Early-Life Social Determinants of SCA6 Age at Onset, Severity, and Progression

**DOI:** 10.1007/s12311-023-01655-w

**Published:** 2024-01-13

**Authors:** Tiffany X. Chen, Hannah L. Casey, Chi-Ying R. Lin, Theresa A. Boyle, Jeremy D. Schmahmann, Gilbert J. L’Italien, Sheng-Han Kuo, Christopher M. Gomez

**Affiliations:** 1 Department of Neurology, Columbia University Medical Center, New York, NY USA; 2 Initiative of Columbia Ataxia and Tremor, Columbia University Medical Center, New York, NY USA; 3 Department of Biomedical Engineering, Johns Hopkins University, Baltimore, MD USA; 4 Department of Neurology, University of Chicago, Chicago, IL USA; 5 Department of Neurology, Baylor College of Medicine, Houston, TX USA; 6 Department of Pathology and Cell Biology, University of South Florida, Tampa, FL USA; 7 Department of Neurology, Massachusetts General Hospital, Ataxia Unit, Cognitive Behavioral Neurology Unit, Laboratory for Neuroanatomy and Cerebellar Neurobiology, Harvard Medical School, Boston, MA USA; 8 Global Health Outcomes and Epidemiology, Biohaven Pharmaceuticals, New Haven, CT USA

**Keywords:** Cerebellar ataxia, Spinocerebellar ataxia, Age of onset, Epidemiology, Cerebellum

## Abstract

SCA6 patients with the same size CAG repeat allele can vary significantly in age at onset (AAO) and clinical progression. The specific external factors affecting SCA6 have yet to be investigated. We assessed the effect of early life events on AAO, severity, and progression in SCA6 patients using a social determinant of health approach. We performed a survey of biological and social factors in SCA6 patients enrolled in the SCA6 Network at the University of Chicago. AAO of ataxia symptoms and patient-reported outcome measure (PROM) of ataxia were used as primary outcome measures. Least absolute shrinkage and selection operation (LASSO) regressions were used to identify which early life factors are predictive of SCA6 AAO, severity, and progression. Multiple linear regression models were then used to assess the degree to which these determinants influence SCA6 health outcomes. A total of 105 participants with genetically confirmed SCA6 completed the assessments. SCA6 participants with maternal difficulty during pregnancy, active participation in school sports, and/or longer CAG repeats were determined to have earlier AAO. We found a 13.44-year earlier AAO for those with maternal difficulty in pregnancy than those without (*p* = 0.008) and a 12.31-year earlier AAO for those active in school sports than those who were not (*p* < 0.001). Higher education attainment was associated with decreased SCA6 severity and slower progression. Early life biological and social factors can have a strong influence on the SCA6 disease course, indicating that non-genetic factors can contribute significantly to SCA6 health outcomes.

**Supplementary Information:**

The online version contains supplementary material available at 10.1007/s12311-023-01655-w.

## Introduction

Spinocerebellar ataxia type 6 (SCA6) is an autosomal dominant, adult-onset neurodegenerative disorder that is characterized by progressive cerebellar ataxia. SCA6 is caused by pathologically expanded CAG repeats in the *CACNA1A* gene. Normal CAG repeat length in the *CACNA1A* gene is up to 18 repeats, whereas individuals with 20 or more CAG repeats will develop ataxia symptoms during a normal lifespan [1]. With 19 CAG repeats, SCA6 has incomplete penetrance with variability in whether SCA6 symptoms occur at all [1]. Although expansions in the *CACNA1A* gene are responsible for SCA6 pathology, CAG repeat length only accounts for 9–41% of variability in the age at onset (AAO) [2, 3]. The disease severity and clinical progression of SCA6 varies substantially between individuals with the same size full-penetrance allele [4]. These observations indicate that other genetic or non-genetic factors may have a significant influence on disease onset and progression in SCA6; however, the specific external factors that contribute to this disease process remain unknown.

Defined as “the conditions in which people are born, grow, work, and age” [5], the social determinants of health are widely regarded as major drivers of health disparities. Life course perspectives further demonstrate that current health is shaped by exposures to physical, environmental, and psychosocial conditions during critical developmental periods. The influence of these early social and environmental factors on monogenetic disorders, such as SCA6, may be important modifiers on the disease course; however, their impact is not well understood. Here, we sought to determine how specific early life conditions and events influence AAO, severity, and progression in SCA6 patients.

## Methods

### Subjects

We performed a survey of early life biological, social, and behavioral factors in a total of 105 SCA6 patients identified through the SCA6 Network at the University of Chicago from January 2021 to February 2022 via consecutive sampling. All cases were 18 years or older with progressive ataxia and a positive genetic test for SCA6. One participant was homozygous for the SCA6 gene (25/25), while all other participants were heterozygous. CAG repeat length was determined based on genetic testing results. Out of the 105 participants, 7 were excluded from the least absolute shrinkage and selection operation (LASSO) regression analysis and AAO regression model due to missing genetic testing results.

### Study Measures

The three health outcomes assessed in this study were AAO, symptom severity, and symptom progression. The AAO was defined as the age at which the first signs of gait impairment appeared*.* Symptom severity was evaluated using the patient-reported outcome measure of ataxia (PROM-Ataxia), which is a 70-item assessment of physical health, activities of daily living, and mental health from the patient’s perspective [6]. Disease progression was calculated using the PROM-Ataxia score over the length of time from AAO.

All patients completed a questionnaire regarding the following early life conditions and events: educational attainment, school sports, traumatic brain injury, maternal difficulty in pregnancy, and recreational drug history. The selection of these conditions is based on the previous association with other neurological disorders [1, 7]. Except for education, all variables were binarized. Levels of educational attainment were stratified into a three-level system as follows: primary education, secondary education, and post-secondary education. Maternal difficulty in pregnancy was defined as experiencing any complications during gestation or labor and delivery, including but not limited to gestational diabetes, hypertension, preeclampsia, and preterm labor.

Participation in school sports was “active” when there was involvement in one or more sports at a community, junior varsity, varsity, or college level. Any incidence of recreational drug use or history of traumatic brain injury was recorded.

### Statistical Analysis

To select for factors predictive of each health outcome in an unbiased manner, we utilized a least absolute shrinkage and selection operation (LASSO) regression. LASSO assesses the contribution of each social determinant surveyed with 500 bootstraps while controlling for sex in the AAO analysis and age and sex in the symptom severity and progression analysis [8]. LASSO regression uses L1 regularization that results in variable selection with high prediction accuracy and specificity of interpretation. Any variable with ≥ 80% chosen is considered significant in relation to AAO, symptom severity, and symptom progression [9]. Multiple linear regression models with control for sex in the AAO analysis and age and sex in the symptom severity and symptom progression analyses were used to measure the strength of association between social determinants found to be predictive by LASSO regression and by each SCA6 health outcome. For each multivariable regression model, multivariate normality was checked by conducting the Shapiro–Wilk test and multicollinearity was checked by determining the variance inflation factors (VIF), with VIF > 10 indicating that independent variables are highly correlated with each other. The threshold for significance was set at 0.05. Multivariable linear and LASSO regression models were performed using the lm and glmnet package respectively and bootstrapped in the R statistical software.

## Results

### Demographics

One hundred five SCA6 patients participated in this study. The average age was 63.15 ± 11.07 years old, and 60% were female. The range of pathological CAG repeat length was 21–27, with a mode of 22. The average AAO for SCA6 symptoms was 43.10 ± 15.97, and the average PROM-Ataxia score was 88.23 ± 54.83. For a summary of the demographic and clinical characteristics of each group, see Table 1. Chi-square analyses were performed to investigate the distribution of early life events according to sex. Interestingly, the frequency of maternal difficulty during pregnancy differed between males and females (1% in males vs. 15% in females, *p* = 0.036, Supplementary Table 1). Pathological CAG repeat length (*p* = 0.608) and disease duration (*p* = 0.236) were not found to be associated with education level through the Kruskal–Wallis test and ANOVA, respectively (Supplementary Table 2).

**Table 1:** Demographic and clinical characteristics of SCA6 participants

Variable	Value
*n*	105
Age	63.15 ± 11.07
Sex (male/female)	42/63
Expanded allele CAG repeat length	22 (21, 27)
Age at onset	43.10 ± 15.97
PROM-Ataxia score	88.23 ± 54.83

### Predictors of Age at Onset

**Fig. 1:**
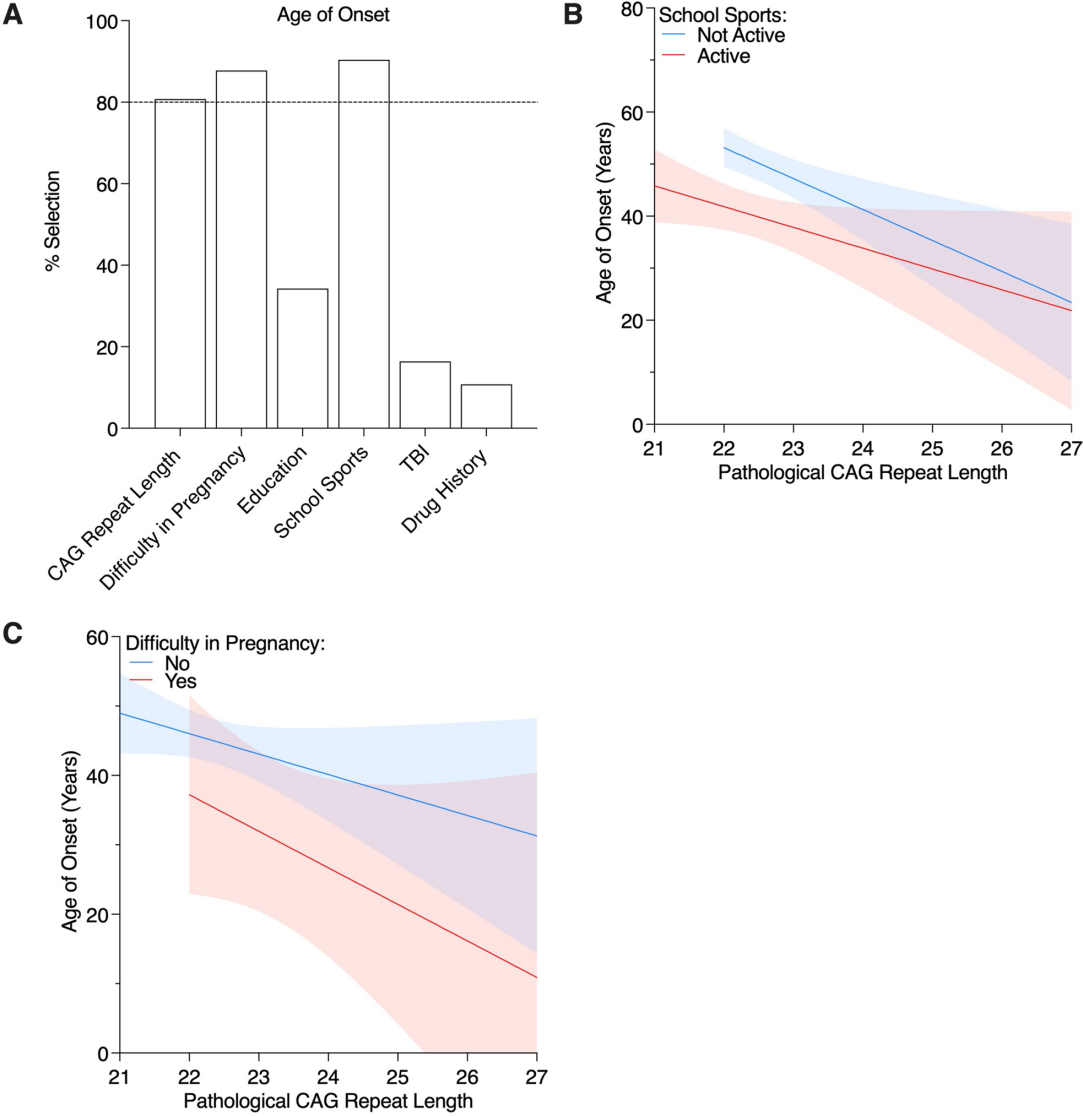
Predictors of age at onset. **A** LASSO selection of the social determinants of age at onset when controlling for sex. The 80% threshold for significant predictive power is shown with the dotted line. Linear regression analysis of age at onset and expanded CAG repeat length as grouped by (**C**) maternal difficulty in pregnancy and (**D**) participation in school sports

We first sought to determine which early life conditions and events influenced the age of symptom onset in SCA6 patients. Through LASSO analysis, three variables with a frequency ≥ 80% were chosen (Fig. 1A): pathological CAG repeat length, participation in school sports, and maternal difficulty in pregnancy. We then constructed a multivariable linear regression model, which included sex and the three LASSO-selected variables, and found that all three of the selected variables remained significant predictors of AAO with an average of a 3.49-year earlier AAO per 1 unit increase of pathological CAG repeat length (95% CI =  − 6.34 to − 0.64, *p* = 0.017), a 13.44-year earlier AAO for those with maternal difficulty in pregnancy than those who did not (95% CI =  − 23.34 to − 3.54, *p* = 0.008), and a 12.31-year earlier AAO for those active in school sports than those who were not (95% CI =  − 18.37 to − 6.24, *p* < 0.001) (Table 2). Notably, the effect sizes of the non-genetic factors were both greater than that of the pathological CAG repeat length. We present visual representations of the effects of genetic and nongenetic influences on AAO in Fig. 1B, C. These results suggest that, in addition to genetic factors, maternal difficulty during pregnancy and active participation in school sports contribute to an earlier age of symptom onset in SCA6 patients.

**Table 2:** Multivariable linear regression model of age at onset of ataxia symptoms using factors selected from LASSO analysis

		Age of symptom onset		
Predictors	*β*	VIF	95% CI	*p*-value
(Intercept)	129.8		(65.74, 193.9)	** < 0.001**
Sex (male)	3.78	1.06	(− 2.09, 9.66)	0.204
Pathological CAG repeat number	− 3.49	1.07	(− 6.34, − 0.64)	**0.017**
Difficulty in pregnancy (yes)	− 13.44	1.04	(− 23.34, − 3.54)	**0.008**
School sports (active)	− 12.31	1.14	(− 18.37, − 6.24)	** < 0.001**
Observations		98		
*R*^2^ Tjur		0.259		
Shapiro–Wilk		0.980, *p* = 0.129		

Bold values denote statistical significance (p < 0.05)

Since previous studies have shown that AAO can be influenced by the size of the normal allele in SCAs [10], we performed an additional multivariable linear regression in order to include the CAG repeat length of the normal allele as another potential predictor of AAO. The results indicated that while the pathological allele length, maternal difficulty during pregnancy, and active participation in school sports remained significant, the normal allele length did not significantly contribute to AAO (*p* = 0.595, Supplementary Table 3).

### Predictors of Disease Severity

**Fig. 2:**
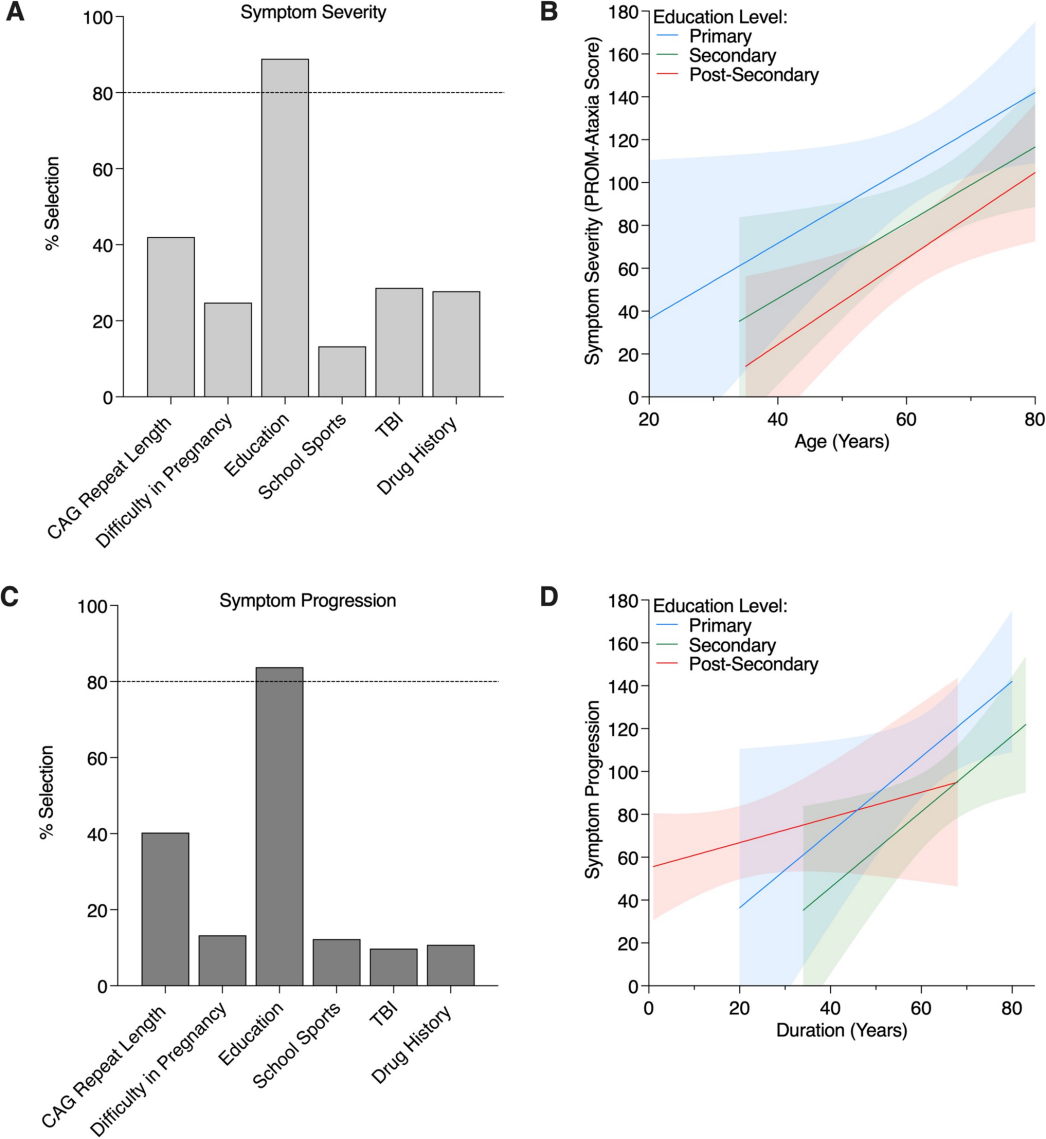
Predictors of symptom severity and progression. LASSO selection of the social determinants of **A** symptom severity and **C** symptom progression when controlling for age and sex. The 80% threshold for significant predictive power is shown with the dotted line. **B** The effects of education on symptom severity are shown by the relationship between age and PROM-Ataxia score. **D** The effects of education on symptom progression are shown by the slope of the regression line

We next queried which early life factors drove disease severity in SCA6 patients. To assess the validity of the PROM-Ataxia as a measure of disease severity in this cohort of SCA6 patients, we determined that PROM-Ataxia scores significantly correlated with disease duration (*r* = 0.236, *p* = 0.016), but not CAG repeat length (*r* =  − 0.007, *p* = 0.944) nor age at onset (*r* = 0.062, *p* = 0.530) using the Spearman correlation. Education level was the only selected variable with a frequency of ≥ 80% with LASSO analysis (Fig. 2A). A linear regression model of disease severity controlling for age and sex indicated that higher levels of educational attainment were significantly predictive of lower symptom severity (Table 3). Specifically, compared to those receiving only primary education, those receiving secondary education were predicted to have a 24.20 lower PROM-Ataxia score on average (95% CI =  − 47.61 to − 0.78; *p* = 0.043) and those receiving post-secondary education were determined to have a 39.95 lower PROM-Ataxia score on average (95% CI =  − 64.01 to − 15.90; *p* < 0.001) (Table 3). A graphical representation of the effects of education level on symptom severity is presented in Fig. 2B. Our findings suggest that higher education levels are associated with lower symptom severity in SCA6 patients.

**Table 3:** Multivariable linear regression models of severity and progression of ataxia symptoms using factors selected from LASSO analysis

	PROM-Ataxia total score				Ataxia disease progression			
Predictors	*β*	VIF	95% CI	*p*-value	*β*	VIF	95% CI	*p*-value
(Intercept)	− 2.48		(− 59.49, 54.54)	0.932	5.35		(− 1.67, 12.4)	0.134
Age	1.88	1.02	(1.03, 2.74)	** < 0.001**	0.06	1.02	(− 0.05, 0.16)	0.268
Sex (male)	− 13.28	1.02	(− 32.47, 5.91)	0.173	0.73	1.02	(− 1.63, 3.09)	0.542
Education (secondary vs. primary)	− 24.20	1.50	(− 47.61, − 0.78)	**0.043**	− 3.06	1.49	(− 5.94, − 0.18)	**0.038**
Education (post-secondary vs. primary)	− 39.95	1.50	(− 64.01, − 15.90)	** < 0.001**	− 3.62	1.50	(− 6.59, − 0.66)	**0.017**
Observations R^2^ Tjur Shapiro–Wilk	105 0.260 0.978, *p* = 0.084				105 0.077 0.862, *p* = 0.133			

Bold values denote statistical significance (p < 0.05)

### Predictors of Disease Progression

Finally, we investigated which early life factors contributed to disease progression in SCA6 patients. LASSO analysis showed that education level was the only selected variable with a frequency of ≥ 80% (Fig. 2C). Using a linear regression model of disease progression controlling for age and sex, higher levels of educational attainment were significantly predictive of slower ataxia progression (− 3.06 PROM-Ataxia score per year for secondary vs. primary education attainment, *p* = 0.038; − 3.62 PROM-Ataxia score per year for post-secondary vs. primary education attainment, *p* = 0.017) (Table 3). The influence of education level on symptom progression is graphically depicted in Fig. 2D. Our results indicate that greater education attainment is associated with slower ataxia progression.

## Discussion

Environmental exposures and life experiences during development have been shown to affect health outcomes in hereditary monogenetic diseases, such as Huntington disease [7]. Currently, there is limited evidence on the mechanisms and degree to which non-genetic factors influence the SCA6 AAO and disease progression rate. In this large SCA6 cohort, we identified specific early-life social determinants that may lead to disparities in AAO, symptom severity, and progression. We found that, in addition to genetic factors, complications in utero and active participation in school sports may contribute to AAO. Our analysis also highlights an education-based disparity in disease severity and progression. Importantly, the study results show that the effect of social determinants on SCA6 health outcomes can be even more significant than CAG repeat length. Further evaluation of the impacts of these non-genetic factors will enhance patient counseling.

Consistent with prior models [11, 12], pathologic CAG repeat length was found to inversely correlate with SCA6 AAO. In this cohort, we found that every 1 unit increase in pathological CAG repeat length was associated with a 3.49 earlier AAO. In comparison, our analysis showed those with maternal difficulties during pregnancy reported a 13.44-year earlier AAO compared to those who did not. This finding is in line with previous studies indicating that prenatal exposures can alter cerebellar development [13, 14]. To determine the specific gestational risk factors that may accelerate the SCA6 disease course, further investigation into maternal environmental exposures and intrinsic factors is warranted.

Although sustained exercise in outpatient rehabilitation settings has been shown to be beneficial in mitigating ataxia disease progression [15], we found active participation in school sports early in life to be associated with an earlier AAO. Among other risks, active participation in contact sports may expose athletes to incidents of repetitive head impacts. Repeat head contacts, even without concussion, have been shown to have a long-term cumulative effect on cortico-cerebellar functioning [16]. Interestingly, the incidence of traumatic brain injury was not found to impact AAO in this study. Taken together, this may suggest that repetitive head injuries at a subclinical level may have a greater influence on SCA6 AAO than a singular high-impact event. However, it should be noted that, here, the type of school sports individuals with SCA6 was not assessed and future studies examining the relationship between school sports and AAO are needed. Additionally, it should be considered that those with active participation in school sports may have noticed signs of disability earlier than those who did not, potentially leading to an earlier AAO.

Variability in SCA6 symptom severity and progression was found to be driven by educational attainment. Specifically, in comparison with those receiving up to primary levels of education, those receiving secondary and post-secondary levels of education were found to have 24.20 and 39.95 points lower PROM-Ataxia scores and slower rate of ataxia progression by 3.06 and 3.62 PROM-Ataxia points per year, respectively. Iannuzzelli et al. similarly found education to be a protective factor in SCA3 progression [17]. Considering that education is often used as a measure for socio-economic status, the positive association between educational attainment and health in individuals with SCA6 may arise from a number of interrelated factors. Higher education levels tend to be predictive of higher incomes, more affluent neighborhoods, and greater access to social and health-related resources with benefits accruing across the life course [5]. Education also enables the development of a broad range of skills and traits that predispose an individual to practice health-promoting behaviors [18]. Additionally, education can be viewed as a proxy for cognitive reserve, and this may indicate that mental stimulation can be beneficial in slowing the SCA6 disease course. Further studies are needed to disentangle the complex relationship between educational attainment and the ataxia disease course.

This study has five main limitations. First, the retrospective design of this study may have limited the detail and scope of the social factors surveyed. Furthermore, retrospective assessment introduces the possibility of recall bias. Future prospective case–control studies are warranted for a more in-depth assessment of primary exposures. Second, we do not have neurologist-rated ataxia severity. However, our study utilized the PROM-Ataxia scale, which is a self-reported outcome measure that reflects the real-life experiences of patients. PROM-Ataxia scores have also been shown to be associated with standard clinical measures of symptom severity, and many ataxic patients are indeed aware of alterations in motor and cognitive functioning [6, 19, 20]. Third, the rate of change in PROM-Ataxia scores was determined based on the time from AAO and assumed to be a linear progression for all patients. Although linear models have been shown to fit progression data across SCAs [21], the relationship between the PROM-Ataxia score and disease duration can vary by patient and additional studies using repeat administration of clinical scales are needed. Fourth, it should be noted that familial ties were present among a small subset of participants, which introduces the possibility of shared biological and environmental factors. Finally, we only studied selected early life determinants, and future studies should expand to explore the influence of more social factors that span throughout the lifetime for a comprehensive assessment.

In conclusion, our study shows that early life biological, behavioral, and social factors can affect the SCA6 disease course and highlights the impact of non-genetic factors on familial degenerative neurological diseases. Future research on the influence of current lifestyle practices and environmental conditions on health in SCA6 patients will help identify present social determinants of SCA6 outcomes.

### Supplementary Information

Below is the link to the electronic supplementary material.

Supplementary file1 (DOCX 35 KB)

## Data Availability

All data generated or analyzed during this study are included in this article and its supplement information files.
