# Supplementary material for: Early-Life Social Determinants of SCA6 Age at Onset, Severity, and Progression
Source: Cerebellum. 2024 Jan 13;23(4):1449–56. doi: 10.1007/s12311-023-01655-w (PMC11269368; doi:10.1007/s12311-023-01655-w)
Supplement: Supplementary file 1 — Supplementary file1 (DOCX 35 KB) [file 12311_2023_1655_MOESM1_ESM.docx]

**SUPPLEMENTARY TABLE**

**Supplementary Table 1.** Absolute and relative frequency distributions of early life events categorized by sex, with a comparison conducted using the Chi-square test.

| **Event** | **Male**  *(n = 43)* | **Female**  *(n = 62)* | ***p*-value** |
| --- | --- | --- | --- |
| TBI (Yes) | 5 (12%) | 10 (16%) | 0.517 |
| School Sports (Active) | 31 (72%) | 38 (61%) | 0.252 |
| Difficulty in Pregnancy (Yes) | 1 (2%) | 9 (15%) | **0.036** |
| Recreational Drug Use (Yes) | 4 (9%) | 3 (5%) | 0.367 |

**Supplementary Table 2.** Comparison of CAG repeat length and disease duration across educational levels using the Kruskal-Wallis test and ANOVA, respectively.

| **Educational Level** | **Pathological CAG Repeat Length**  (Median, Range) | **Disease Duration**  (Mean ± Std Dev) |
| --- | --- | --- |
| Primary (*n* = 28) | 22, [22, 25] | 18.18 ± 11.20 |
| Secondary (*n* = 40) | 22, [22, 25] | 21.78 ± 15.27 |
| Post-Secondary (*n* = 37) | 22, [21, 27] | 20.63 ± 18.20 |
| *p*-value | 0.608 | 0.236 |

**Supplementary Table 3.** Multivariable linear regression model of age at onset of ataxia symptoms using factors selected from LASSO analysis and the normal CAG repeat number.

|  | **Age of Symptom Onset** | | | | | |
| --- | --- | --- | --- | --- | --- | --- |
| **Predictors** | **β** | | | **VIF** | **95% CI** | ***p*-value** |
| (Intercept) | | 148.4 | |  | [68.74, 228.4] | **<0.001** |
| Sex (Male) | | 2.80 | | 1.12 | [-3.63, 9.23] | 0.389 |
| Pathological CAG Repeat Number | | -4.52 | | 1.18 | [-8.12, -0.93] | **0.014** |
| Difficulty in Pregnancy (Yes) | | -14.13 | | 1.25 | [-25.23, -3.02] | **0.013** |
| School Sports (Active) | | -12.10 | | 1.07 | [-18.76, -5.43] | **<0.001** |
| Normal CAG Repeat Number | | 0.40 | | 1.09 | [-1.10, 1.91] | 0.595 |
| Observations | |  | 88 | | | |
| R^2^ Tjur |  | | 0.263 | | | |
| Shapiro-Wilk |  | | 0.980, *p =* 0.184 | | | |
